# Predicting Human Protein Subcellular Locations by the Ensemble of Multiple Predictors via Protein-Protein Interaction Network with Edge Clustering Coefficients

**DOI:** 10.1371/journal.pone.0086879

**Published:** 2014-01-23

**Authors:** Pufeng Du, Lusheng Wang

**Affiliations:** 1 School of Computer Science and Technology, Tianjin University, Tianjin, China; 2 Department of Computer Science, City University of Hong Kong, Kowloon, Hong Kong; Nazarbayev University, Kazakhstan

## Abstract

One of the fundamental tasks in biology is to identify the functions of all proteins to reveal the primary machinery of a cell. Knowledge of the subcellular locations of proteins will provide key hints to reveal their functions and to understand the intricate pathways that regulate biological processes at the cellular level. Protein subcellular location prediction has been extensively studied in the past two decades. A lot of methods have been developed based on protein primary sequences as well as protein-protein interaction network. In this paper, we propose to use the protein-protein interaction network as an infrastructure to integrate existing sequence based predictors. When predicting the subcellular locations of a given protein, not only the protein itself, but also all its interacting partners were considered. Unlike existing methods, our method requires neither the comprehensive knowledge of the protein-protein interaction network nor the experimentally annotated subcellular locations of most proteins in the protein-protein interaction network. Besides, our method can be used as a framework to integrate multiple predictors. Our method achieved 56% on human proteome in absolute-true rate, which is higher than the state-of-the-art methods.

## Introduction

A cell is deemed to be the most basic construction unit of every living creature on this planet. Every living cell is composed of even more basic components, which are known as the subcellular compartments or subcellular organelles [1]. In general, there are two types of subcellular organelles, the membrane bounded subcellular compartments and the non-membrane bounded subcellular structures. The membrane bounded subcellular compartments can be roughly considered as isolated spaces surrounded by bio-membranes. For example, the mitochondria, the cell nucleus and the chloroplasts in green plants are all membrane bounded subcellular organelles. Some other subcellular structures, such as the ribosomes, the cytoskeleton and the centriole, which are non-membrane bounded, may also be recognized as subcellular organelles [2]. All these subcellular structures form a large dynamic system within a cell. The proteins and other macromolecules are synthesized, transferred and activated for their function within this system [3].

Changes in protein location are associated with a host of genetic disorders [4]. For example, the STAT3 (Signal Transducer and Activator of Transcription 3) should be directed to the nucleus in normal cells, while inappropriate nuclear relocation of STAT3 promotes oncogenesis through abnormal cell cycle progression, angiogenesis, and invasion of tissue [5]. Another example was in Zellweger syndrome. The mis-location of some peroxisomal proteins leads to dysfunctional fatty acid oxidation [6]. A third example was in glioma. A recent study showed that the GFRA4 (GDNF Family Receptor Alpha 4) are mis-located in the glioma. The artificial redirection of GFRA4 to the correct target results in a dramatic decrease in proliferation of glioma cells [7]. Therefore, the knowledge of accurate protein subcellular locations is of fundamental importance to both the life science and the drug industry.

There are several experimental methods that can determine the protein subcellular locations. For example, in yeast, the subcellular location of proteins can be visualized systematically by fusion of each ORF (Open Reading Frame) to the gene encoding GFP (Green Fluorescent Protein), either through transposon mutagenesis or PCR (Polymerase Chain Reaction) tagging [8], [9]. This technology requires the analysis of images, where a fully automated procedure is still not readily available [10]. Moreover, this technology is hardly feasible in humans and other mammals. In these organisms, immunolabeling and cell fractionation followed by tandem mass spectrometry were commonly applied [11], [12].

Unfortunately, all these experiments are costly and time consuming [13]. With the progress of proteome projects of many organisms, the number of known protein sequences has increased exponentially in the last two decades [14]. Experimental annotation of protein subcellular locations is too slow to catch up with the increment of protein sequences. A huge information gap between the protein sequences and their annotations has been created. Moreover, this gap is becoming wider with each passing day. To bridge this gap, many computational methods have been developed in the past few years to predict the protein subcellular locations from the primary sequences. These sequence-based methods generally fall into two categories: the signaling peptides based methods and the pseudo-amino acid composition based methods.

According to the cell biology, the proteins are usually synthesized in the cytosol and are transported to other subcellular compartments either during or after the translation [15]. The targets of the transportation are determined by the signaling peptides, which are short peptides that mostly reside in the N-terminus of protein sequences [15]. In some cases, these signals can also reside in the other parts of protein sequences. For example, the PTS1 (Peroxisomal Targeting Signal 1) peptides, which direct proteins to peroxisome, reside in the C-terminus of protein sequences [16]. If the signaling peptides can be found in the protein sequences, they can be used to predict their subcellular locations. Many impressive achievements have been made by finding the signaling peptide on the protein sequences [17]–[21].

However, due to the limitation of protein sequencing technology, the accuracy of the N-terminus of a protein sequence is not ideal, which restricted the application of signaling peptides based methods [22]. On the other hand, the subcellular location of a protein actually provides a micro physicochemical environment that should be compatible with the average physicochemical properties of a proteins surface [23], which was found to be related to the amino acid composition of a protein sequence [24]. Therefore, a large number of efforts have been made to predict the protein subcellular locations by using the pseudo-amino acid composition, which can be recognized as a universal numerical representation of the entire protein sequence [25], [26]. Several recent reviews have summarized the representative studies of this kind [27]–[29].

Rather than the above two sequence-based categories of methods, a number of sequence-based meta-predictors have been developed in the last few years. These works focused on developing voting schemes to combine the results of existing sequence-based predictors. Liu et al. proposed a weighted and adaptive voting scheme to integrate the prediction results of twelve independent predictors [30]. Laurila and Vihinen proposed the PROLocalizer method to combine over a dozen predictors based on signaling peptides analysis [31]. Park et al. developed an LDA (Linear Discriminative Analysis) based voting scheme to combine thirteen predictors [32]. Lin et al. proposed a minimalist ensemble algorithm that combined four predictors [33]. Magnus et al. proposed a voting scheme to combine four predictors for predicting protein subcellular locations in gram-negative bacteria [34]. By making use of the prediction results of existing predictors, these methods are different to the traditional ensemble classifiers, which create every module classifier in the ensemble [35]–[39].

From the system biology point of view, the proteins within a cell do not work independently. They interact with different proteins under different conditions. Because the physical interactions between a couple of proteins actually implied that the physical distance between interacting proteins is very close, the interacting proteins tend to localize within the same subcellular compartments [40], [41]. Furthermore, some proteins that lack of proper signaling peptides may be directed to its destination by a piggy-back mechanism, in which the signaling peptide is contained by the interacting partners of the protein instead of the protein itself [42], [43]. These facts implied that the protein-protein interaction information should be useful in predicting protein subcellular locations.

Several methods have been developed based on the protein-protein interactions in predicting protein subcellular locations. Scott et al. integrated protein-protein interaction as a module in their PSLT2 method to analyze the subcellular location in proteome-wide in yeast [44]. Lee et al. hybridized a group of network based features with pseudo-amino acid compositions in predicting protein subcellular locations [45]. Shin et al. developed a method to predict the protein subcellular locations from its interacting partners [46]. Mintz-Oron et al. used metabolic networks for enzyme localization prediction using constraint-based models [47]. Kumar and Ranganathan used statistical tests to analyze whether the interacting proteins would co-localize in both protein-protein interaction network and the metabolite-linked protein interaction network [48]. Jiang and Wu compared the performances of several different methods using protein-protein interaction networks and developed an ensemble classifier that can better identify subcellular locations on yeast protein-protein interaction network [49]. Mondal and Hu proposed the NetLoc method that can predict protein subcellular locations using four different types of protein networks [50]. In [45], [49], statistical inference based methods were employed to define a parameter for every interaction.

For almost all the existing methods, the protein-protein interaction network was used either as an independent predictor [44]–[46] or as a module classifier, whose results were further utilized in an ensemble [33]. Most of the existing studies require a complete and accurate protein-protein interaction network as the foundation of their method, and the experimental subcellular location annotations of most proteins in the protein-protein interaction network were usually required to improve the prediction performance.

In this paper, we propose to use the protein-protein interaction network as an infrastructure to integrate other sequence based predictors. The results of sequence-based predictors were combined on the protein-protein interaction network. When predicting the subcellular locations of a given protein, not only the protein itself, but also all its interacting partners were considered. These interacting partners were not treated equally, as a weight parameter was given to every interaction between the given protein and each partner. Unlike existing methods, our method does not require the comprehensive knowledge of the protein-protein interaction network. Moreover, given a protein, our method does not require the experimentally annotated subcellular locations of all its neighbors to predict its subcellular locations.

These characteristics of our method makes it possible to work on an incomplete and inaccurate protein-protein interaction network with only limited number of proteins annotated with subcellular locations. Besides, our method can be used as a framework to integrate multiple predictors. We demonstrate that, in human proteome, even with only one sequence based predictor, our method can improve its prediction performance with the help of protein-protein interaction data. Therefore, we can expect that our method can improve the prediction performance of most existing sequence-based predictors.

## Materials and Methods

### 1 Protein-protein interactions dataset

The protein-protein interaction data were retrieved from BioGRID database version 3.2.96. The following filtering steps were carried out. (1) Only interactions between two human proteins were kept. (2) The interactions between two identical proteins were removed. (3) If some interactions appeared more than once in the dataset, only one interaction was kept. (4) The non-physical interactions were removed. After these filtering steps, there were 96967 interactions covering 13942 proteins remaining in the dataset. The dataset of this study can be obtained from the authors by email.

### 2 Experimental subcellular location annotations

The 13942 proteins were mapped to the UniProt database version 2013_07. These proteins in the protein-protein interaction network can be mapped to 18036 proteins in the UniProt database. The subcellular location annotations of these 18036 proteins were collected. If an annotation was marked as “Probable”, “By Similarity” or “Potential”, this annotation was discarded. The remaining subcellular location annotations were mapped to 11 different terms, including “Cell membrane”, “Cytoplasm”, “ER”, “Extracell”, “Golgi”, “Mitochondrion”, “Nucleus”, “Peroxisome”, “Lysosome”, “Endosome” and “Microsome”. The mapping was carried out following the keyword searching strategy as the state-of-the-art studies [51], [52]. Any subcellular location annotations that cannot be mapped to these terms were discarded.

**Figure 1:**
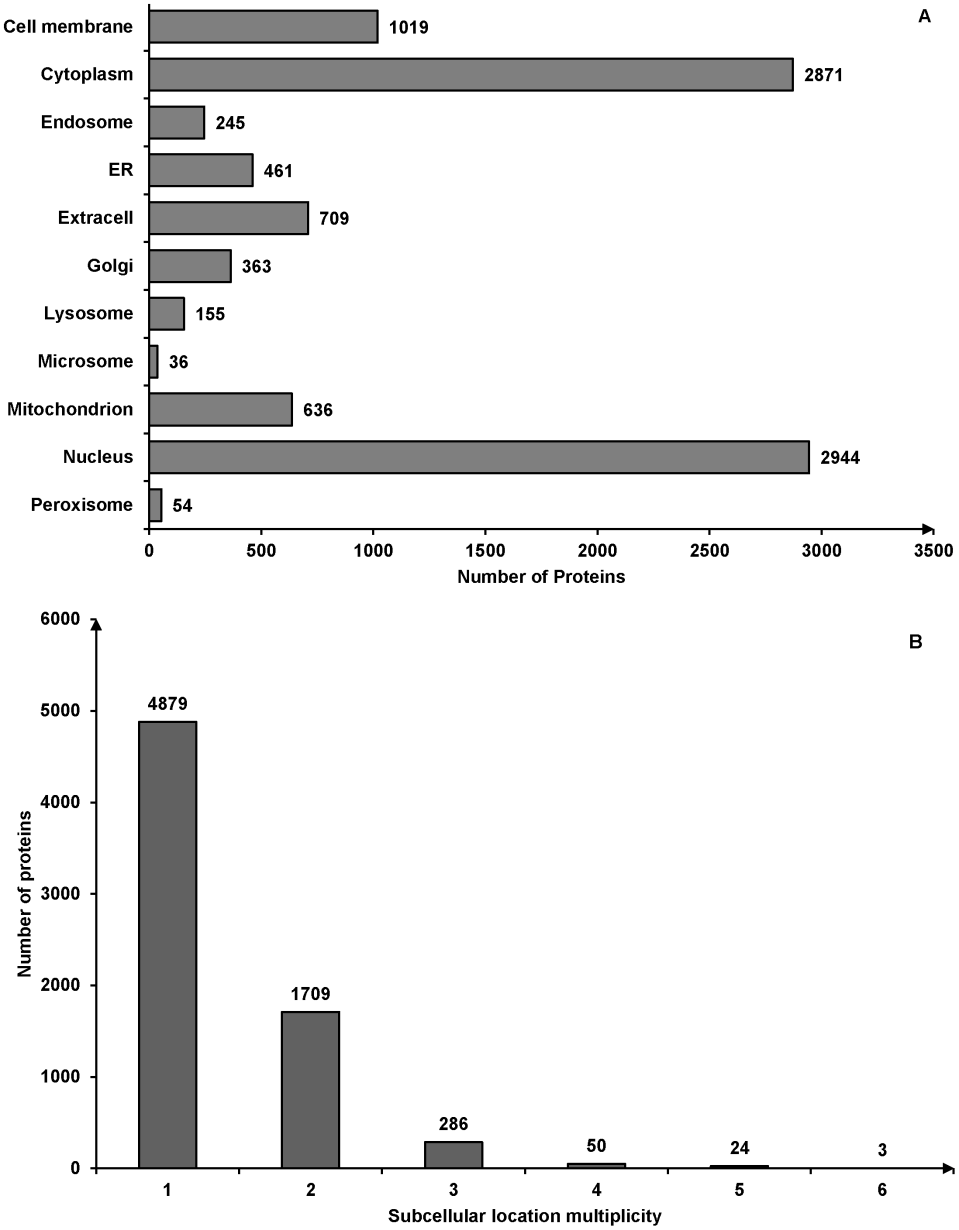
The summary of dataset. (A) The number of locative proteins in different subcellular locations. There are 6951 proteins with experimentally annotated subcellular locations in the dataset. Because one protein may have more than one subcellular location, the number of locative proteins is 9493. (B) The number of proteins with different number of subcellular locations.

To avoid ambiguous descriptions, we termed the 13942 proteins as the “BioGRID proteins” and the 18036 proteins as the “UniProt proteins”. If a BioGRID protein can be mapped to one or more than one UniProt proteins, the subcellular location of this BioGRID protein is the collection of all subcellular locations of all mapped UniProt proteins. Otherwise, the subcellular location of this BioGRID protein is “Unknown”. There were 6951 BioGRID proteins that can be annotated from the above procedure. Among 6951 proteins, there were 4879 proteins with only one subcellular location, 1709 proteins with two locations, 286 proteins with three locations, 50 proteins with four locations, 24 proteins with five locations and 3 proteins with six locations. According to the locative protein concept [52], this created 9493 locative proteins. The average multiplicity degree of the dataset was 1.37 [53]. The breakdown of the dataset in both the subcellular location multiplicity and the subcellular locations types can be found in Figure 1. According to these data, only about half proteins in the protein-protein interaction network have experimentally annotated subcellular locations. We used the results of sequence based predictors as the complementary and enhancements to the experimental annotations.

### 3 Sequence based subcellular location predictions

There are a number of existing methods that can predict protein subcellular locations based on primary sequences. We find that the Hum-mPLoc 2.0 [36] and Y-Loc [54] predictors provided the most convenient and reliable services that are suitable for our work. Therefore, two sequence based predictors were integrated in this work, Hum-mPLoc 2.0 [36] and Y-Loc [54]. According to Lin et al. [33], the integrated predictors should cover as many types of features as possible to improve the prediction performance in a meta-predictor [33]. These two predictors covered the pseudo-amino acid compositions, gene ontology annotations, evolutionary features and the signaling peptides features. Both predictors provided prediction results on the UniProt proteins, regardless to whether they have been experimentally annotated in the database. The subcellular location predictions of the BioGRID proteins were generated based on the results of these two predictors, respectively, as if they provided experimental annotations. If a predictor did not provide any prediction result for a protein, the result of this protein was recorded as “Unknown”. As the subcellular location terms in the two predictors are not identical to the 11 subcellular locations in this study, their location terms were mapped to the 11 subcellular location terms according to the biological definitions and the UniProt-GOA mapping [55].

### 4 Edge clustering coefficients

As indicated by existing studies, the reason why physical protein-protein interactions can be used to predict protein subcellular locations is that the physical locations of two interacting proteins are very close, which make them tend to localize within the same subcellular organelle [45], [46]. However, given two interacting proteins, it is difficult to infer that whether two proteins would have common subcellular locations directly from the protein-protein interaction network without knowing the subcellular locations of either protein. Fortunately, we find that ECC (Edge Clustering Coefficient) can be used as an indicator of whether two interacting proteins tend to have common subcellular locations.

ECC, which was originally proposed in the analysis of social networks [56], was employed in this study as an indicator of whether two interacting proteins tend to have common subcellular locations. According to Wang et al. [57], ECC can be used to describe the importance of an protein-protein interaction, as well as how close two interacting proteins are [57]. It had achieved many success in identifying essential proteins and protein complexes [57]–[59]. The definition of ECC can be described as follows.

For an interaction between two proteins *p_i_* and *p_j_*, the ECC of this interaction can be defined as follows:
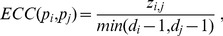
(1)


where *z_i,j_* is the number of triangles that actually involve the edge connecting *p_i_* and *p_j_* in the network, *d_i_* and *d_j_* the degrees of protein *p_i_* and *p_j_* in the network, respectively. The denominator means the number of triangles in which the edge connecting *p_i_* and *p_j_* may possibly participate at most.

### 5 Network based meta-predictor

Before we describe our network based method, we define some notations as follows. Let *G* =  (*V*, *E*) be a PPI network, where *V* is the set of *n* vertices and *E* is the set of edges. Each vertex represents a protein (i.e. *V* = {*p_1_*, *p_2_*, …, *p_n_*}) and an edge *E_i,j_* indicates that protein *p_i_* and protein *p_j_* has a physical interaction.

For every protein *p_i_*∈*V* (*i* = 1, 2, …, *n*), there are *m* possible subcellular locations (i.e. *L* = {*l_1_*, *l_2_*, …, *l_m_*}). In the current study, *m* = 11. Every protein can have one or more subcellular locations. The set of experimentally determined subcellular location of *p_i_* was denoted as *SCL*(*p_i_*). The set of Hum-mPLoc 2.0 predicted subcellular locations of *p_i_* was denoted as *MP*(*p_i_*). The set of Y-Loc predicted subcellular locations of *p_i_* was denoted as *YC*(*p_i_*). They are all subsets of *L* (i.e. *SCL*(*p_i_*) ⊆ *L*, *MP*(*p_i_*) ⊆ *L* and *YC*(*p_i_*) ⊆ *L*). If the experimental subcellular location does not exists, *SCL*(*p_i_*) = ∅. If the Hum-mPLoc 2.0 or Y-Loc cannot provide subcellular locations for protein *p_i_*, *MP*(*p_i_*)  = ∅ or *YC*(*p_i_*)  = ∅.

We defined the set of proteins with subcellular location *l_k_* (*k* = 1, 2, …, *m*) in experimental annotations (*S_k_*(*SCL*)), Hum-mPLoc 2.0 predictions (*S_k_*(*MP*)) or Y-Loc predictions *S_k_*(*YC*) as:
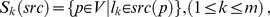
(2)


where *src* ∈ {*SCL*, *MP*, *YC*}.

With all the above definitions, we now describe the network based method for predicting protein subcellular locations. Given a protein, its subcellular locations will be predicted based on the experimental and predicted subcellular locations of its interacting partners as well as the interactions between them. The sequence-based predictors can also provide predictions directly to the given protein. Our method considered all the above information to make the final predictions.

For every protein *p_u_*∈*V*, which has no experimental subcellular locations, we use the following steps to predict its subcellular locations.

Step 1: we find its neighbors in the PPI network. The set of the neighbors was denoted as *NE*(*p_u_*). We calculate the probability that observing any member of *S_k_*(*src*) in *NE*(*p_u_*) as follows:
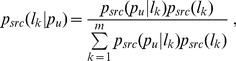
(3)


where 
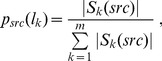
(4)

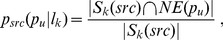
(5)


*src* ∈ {*SCL*, *MP*, *YC*}, and |.| the cardinal of a set.

Step 2: the membership degree of a protein *p_u_* to a subcellular locations *l_k_* can be computed as 
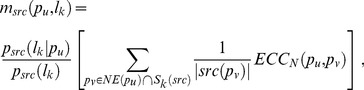
(6)


where
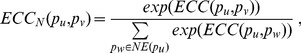
(7)


*exp*(.) the exponential function, and *src* ∈ {*SCL*, *MP*, *YC*}. Here *ECC_N_*(*p_u_*, *p_v_*) ≠ *ECC_N_*(*p_v_*, *p_u_*), as we found that *ECC_N_*(*p_u_*, *p_v_*) can achieve better performance than *ECC*(*p_u_*, *p_v_*).

Step 3: for every *src* ∈ {*SCL*, *MP*, *YC*}, a set of subcellular locations can be predicted for protein *p_u_*, as follows:
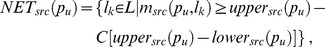
(8)


where
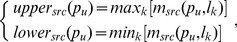
(9)


*NET_src_*(*p_u_*) the set of predicted subcellular locations and *C* a parameter between 0 and 1.

In addition, if the following condition cannot be satisfied, we forced that *NET_src_*(*p_u_*)  = ∅.
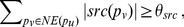
(10)


where *θ_src_* is an integral parameter for every *src*∈{*SCL*, *MP*, *YC*}.

Step 4: the previous step provided three predictions: *NET_SCL_*(*p_u_*), *NET_MP_*(*p_u_*) and *NET_YC_*(*p_u_*). The sequence based predictors can also provide direct predictions on *p_u_*, as *MP*(*p_u_*) and *YC*(*p_u_*). We defined the following three sets *SEQ*(*p_u_*), *NET_SEQ_*(*p_u_*) and *NET*(*p_u_*):
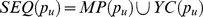
(11)

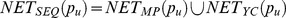
(12)

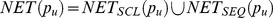
(13)


The meaning of these definitions can be explained as follows. The *SEQ*(*p_u_*) is the prediction results from sequence information directly. The *NET_SEQ_*(*p_u_*) is the prediction results from network information with only the predicted subcellular location of the neighborhood proteins. The *NET*(*p_u_*) is also the prediction results from network but with both the predicted subcellular locations and the experimental subcellular locations of the neighborhood proteins.

For every *l_k_*∈*L*, *l_k_* belongs to the final predictions if and only if it satisfy either of the following two conditions: (1) *l_k_*∈*NET*(*p_u_*)∩*SEQ*(*p_u_*); (2) *m_src_*(*p_u_*, *l_k_*)  =  *upper_src_*(*p_u_*, *l_k_*) for all *src*∈ {*SCL*, *MP*, *YC*}. This can be represented as follows:
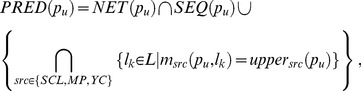
(14)


where *PRED*(*p_u_*) is the final prediction results. When *PRED*(*p_u_*)  = ∅, we used *MP*(*p_u_*) as *PRED*(*p_u_*), as Hum-mPLoc 2.0 performs better than Y-Loc.

### 6 Parameter calibrations

In eqn (8) and eqn (10), there are several parameters. We used a grid search method to find the best combination of these parameters. The parameter *C* in eqn (8) was searched from 0.5 to 0.95 with step 0.05. The parameter *θ_SCL_* was searched from 1 to 20 with step 1. The parameter *θ_MP_* and *θ_YC_* was searched from 10 to 100 with step 10. Altogether 2000 trials were carried out. We finally achieved an optimized combination when *C* = 0.75, *θ_SCL_* = 1 and *θ_MP_* = *θ_YC_* = 60.

### 7 Performance evaluations

Jackknife test has been widely used by many investigators to examine the quality of various predictors, as summarized in a recent review [27]. In this study, we also used jackknife test to evaluate the performance of our method. Because every protein may have one or more subcellular locations, using the traditional performance measures is difficult [53]. To measure the performance of a multi-label predictor, a set of statistical measures was established [53], [60], [61]. These statistical measures include aiming (*AIM*), coverage (*CVR*), accuracy (*ACC*), absolute-true rate (*ATR*) and absolute-false rate (*AFR*). They can be formulated as follows:
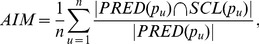
(15)

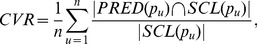
(16)

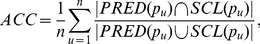
(17)




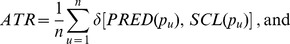
(18)

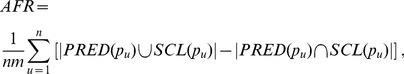
(19)


where *n* and *m* are the total number of proteins and subcellular locations respectively, and



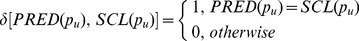
(20).

These measures can be interpreted briefly here. The *AIM* [53], which is also called “Precision”[60] or “Positive Predictive Value”[61], reflects the average ratio of correctly predicted subcellular locations over all predicted locations. The *CVR* [53], which is also termed as “Recall” [60] or “Sensitivity”[61], reflects the average ratio of the correctly predicted subcellular locations over the real locations. The *ACC* reflects the average ratio of correctly predicted subcellular locations over the total locations including the predicted and the real ones [53], [60], [61]. The *ATR* [53], [61], which is also called “Subset-accuracy”[60], reflects the ratio of proteins without either over-predicted locations or under-predicted locations. The *AFR* [53], which is also termed as “Hamming-Loss”[60], [61], is the average ratio of over-predicted locations and under-predicted locations over the total number of possible locations. Unlike the previous four measures, which are all the higher the better, a lower *AFR* value indicates a better prediction performance. A more comprehensive discussion of these measures can be found in several reviews [53], [60], [61].

The performance measures, which were utilized by Hum-mPLoc 2.0, were based on the locative protein concept [36]. However, according to a recent review [53], the prediction performance for each subcellular location based on locative protein concept is inconsistent with the *ATR* measure [53]. Therefore, we only use the multi-label performance measures, such as *AIM*, *CVR* and *ATR*, as the performance measures in our study.

## Results and Discussion

### 1 Correlation between ECC and common subcellular locations

Given two protein *p_i_* and *p_j_*, which are connected by an edge *E_ij_* in the protein-protein interaction network, we defined the co-localization score (*Q_ij_*) as follows: 
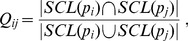
(21)


where *SCL*, *p_i_* and *p_j_* have the same meanings as in the Method section. The numerator of eqn (21) is the number of subcellular locations that *p_i_* and *p_j_* both localized to. The denominator of eqn (21) is the number of subcellular locations that at least one of *p_i_* and *p_j_* localized to. When *p_i_* and *p_j_* have identical subcellular locations, *Q_ij_* = 1. When *p_i_* and *p_j_* have no common subcellular location, *Q_ij_* = 0. When *p_i_* and *p_j_* have some common subcellular locations, but not identical subcellular locations, the value of *Q_ij_* indicates the fraction of the number of common subcellular locations over the total number of subcellular locations of *p_i_* and *p_j_* (i.e. 0 < *Q_ij_* < 1). Therefore, *Q_ij_* can indicate whether *p_i_* and *p_j_* tend to have the same subcellular locations. When *p_i_* or *p_j_* have no subcellular location annotations, *Q_ij_* cannot be computed.

**Figure 2:**
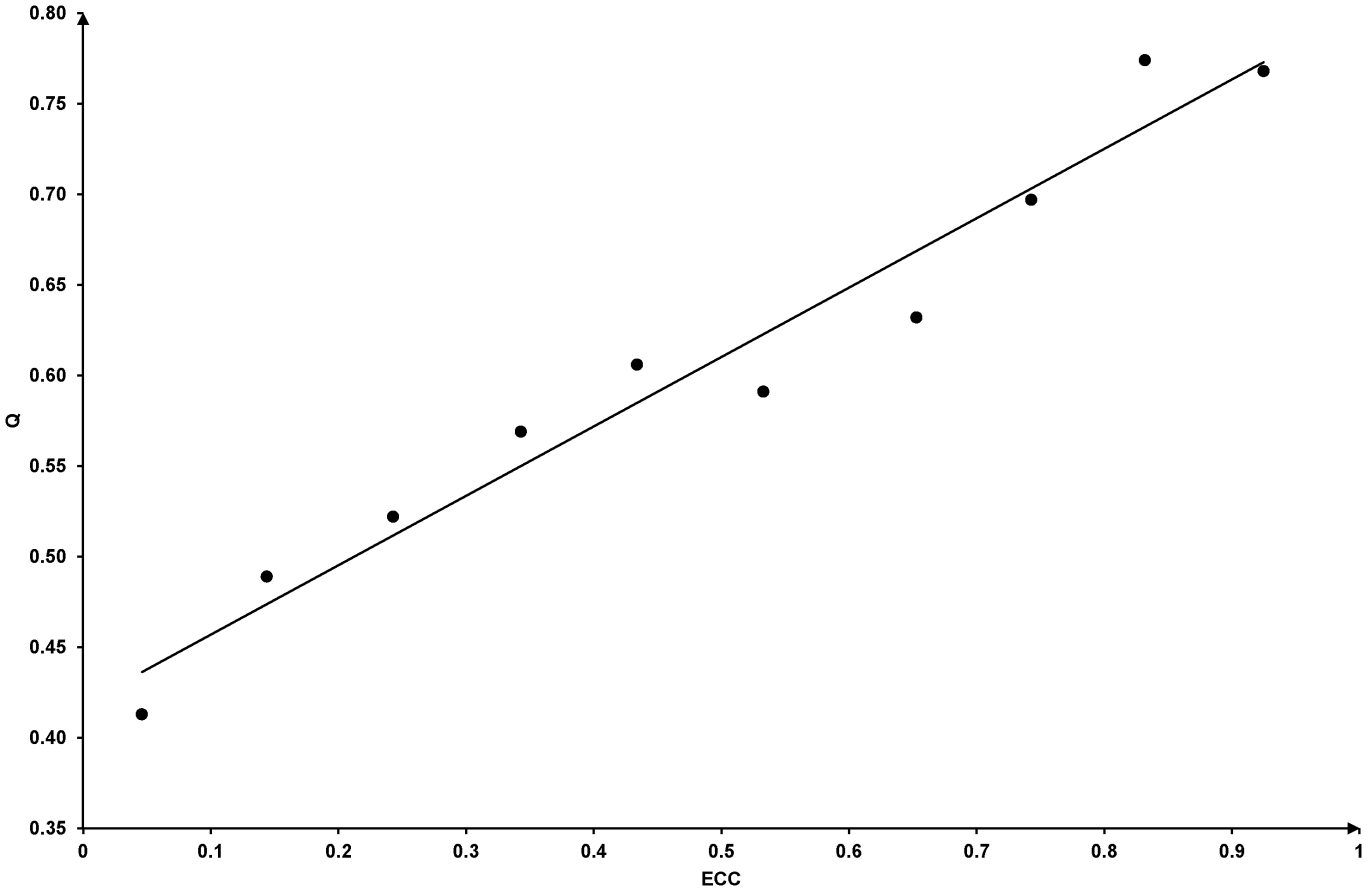
The relationship between ECC and co-localization scores. For every pair of interacting proteins with experimentally annotated subcellular locations, the ECC of their interactions and the co-localization score were computed. These interactions were divided into ten groups according to their ECC values. The first group contained the interactions with ECC value between 0 and 0.1.The second group contained the interactions with ECC value between 0.1 and 0.2. The third group contained the interactions with ECC value between 0.2 and 0.3, and so forth. The average values of ECC and co-localization score were computed for every group. The horizontal axis of this figure is the average value of ECC. The vertical axis of this figure is the average value of co-localization score. Ten dots were plotted on this figure to represent the ten groups of interactions. A straight line was generated using simple linear regression method to represent the linear relationship between the average ECC and the average co-localization score.

For every edge *E_ij_* that connects two proteins with subcellular location annotations, a *Q_ij_*, as well as an *ECC*(*p_i_*,*p_j_*), can be computed. We plotted the average *Q_ij_* as a function of the average *ECC*(*p_i_*,*p_j_*) in different ranges, such as [0,0.1), [0.1,0.2), …, [0.9,1.0). As shown in Figure 2, when 0≤*ECC*(*p_i_*,*p_j_*)<0.1, the average *Q_ij_* is about 0.41. When 0.1≤*ECC*(*p_i_*,*p_j_*)<0.2, the average *Q_ij_* is about 0.48. The average *Q_ij_* continues to increase along with the ECC. When 0.9≤*ECC*(*p_i_*,*p_j_*)<1, the average *Q_ij_* reaches 0.77. When *ECC*(*p_i_*,*p_j_*) varies in different ranges, the linear correlation coefficient between the average *Q_ij_* and the average *ECC*(*p_i_*,*p_j_*) is 0.96.

With the above observations, it is reasonable to use ECC as an indicator to whether two interacting proteins tend to have common subcellular locations.

### 2 Performance analysis

The prediction performance of our method was estimated using jackknife test on our dataset. In order to compare prediction performances, the performance of Hum-mPLoc 2.0 and Y-Loc was also estimated on the same dataset.

As shown in Table 1, the *ATR* of our method achieves 56.0%, while the *ATR* of Hum-mPLoc 2.0 is 51.4% and Y-Loc 47.4%. The *ACC* of our method achieves 70.0%, while the *ACC* of Hum-mPLoc 2.0 is 67.1% and Y-Loc 59.8%. The only measure that our method is slightly lower than Hum-mPLoc 2.0 is the *CVR*. Our method is 74.9% in *CVR* and Hum-mPLoc 2.0 is 75.4%. Since the *ATR* is the most strict and harsh measure of a predictor that deals with multi-label data [53], [62], the prediction performance of our method is better than both integrated methods.

**Table 1:** Comparison of prediction performances.

Predictor	AIM^a^	CVR^b^	ACC^c^	ATR^d^	AFR^e^
Hum-mPLoc 2.0	75.7%	75.4%	67.1%	51.4%	7.4%
Y-Loc	72.4%	61.0%	59.8%	47.4%	8.4%
This method	79.8%	74.9%	70.0%	56.0%	6.5%

a AIM is Aiming, as defined in eqn (15);

b CVR is Coverage, as defined in eqn (16);

c ACC is Accuracy, as defined in eqn (17);

d ATR is Absolute-True-Rate, as defined in eqn (18);

e AFR is Absolute-False-Rate, as defined in eqn (19).

### 3 Examples of better predictions

Here, we provide some examples that our method gives better predictions than Hum-mPLoc 2.0. Because when our method cannot make predictions based on network information, we used the Hum-mPLoc 2.0 predictions as the final results, it is important to look into the details that how our method use network information to improve the prediction results of Hum-mPLoc 2.0.

The first example is the BioGRID protein 107454. It can be mapped to UniProt protein P11802, which has two experimentally annotated subcellular locations, “Cytoplasm” and “Nucleus”, in the UniProt database. Hum-mPLoc 2.0 provided only one result “Nucleus” based on sequence information. Our method, based on the network information, supplied the “Cytoplasm” location.

The second example is the BioGRID protein 107479. It can be mapped to UniProt protein P49715, which has only one experimentally annotated subcellular location, “Nucleus”, in the UniProt database. Hum-mPLoc 2.0 provided two subcellular location predictions, including “Cytoplasm” and “Nucleus”. Our method, based on the network information, removed the “Cytoplasm” location.

The third example is the BioGRID protein 107693. It can be mapped to UniProt protein P25067, which has only one experimentally annotated subcellular location, “Extracell”, in the UniProt database. However, Hum-mPLoc 2.0 provided “Cytoplasm” as its prediction result. Our method, based on the network information, corrected this result to “Extracell”.

There are a number of examples like the above three that our method actually provided better predictions than Hum-mPLoc 2.0. These better predictions can be achieved by supplying the extra predictions, removing the redundant predictions or correcting the wrong predictions.

### 4 Improving the prediction performance of single sequence based predictor

In the above results, we integrated two sequence based predictors. Actually, our method can work with only one sequence based predictor. Without optimizing the parameters, we directly applied our method with only Y-Loc predictor. The *ATR* is 48.6%, which is higher than the 47.4% of Y-Loc predictor independently. Again, without optimizing parameters, we directly applied our method with only Hum-mPLoc 2.0. Our method can achieve 54.9% in *ATR*, which is also higher than the 51.4% of Hum-mPLoc 2.0 predictor. A comprehensive performance can be found in Table 2. These results imply that our method can be used as a common approach to improve most existing sequence based predictors.

**Table 2:** Performance improvements for every single predictor.

Predictor	AIM^a^	CVR^b^	ACC^c^	ATR^d^	AFR^e^
Hum-mPLoc 2.0	75.7%	75.4%	67.1%	51.4%	7.4%
Hum-mPLoc 2.0 + PPI^f^	79.1%	72.0%	68.4%	54.9%	6.8%
Y-Loc	72.4%	61.0%	59.8%	47.4%	8.4%
Y-Loc + PPI^g^	73.2%	61.1%	60.5%	48.6%	8.2%

a AIM is Aiming, as defined in eqn (15);

b CVR is Coverage, as defined in eqn (16);

c ACC is Accuracy, as defined in eqn (17);

d ATR is Absolute-True-Rate, as defined in eqn (18);

e AFR is Absolute-False-Rate, as defined in eqn (19);

f These performance values were obtained without optimizing parameters. “+PPI” means using the current method with only Hum-mPLoc 2.0;

g These performance values were obtained without optimizing parameters. “+PPI” means using the current method with only Y-Loc.

### 5 Iterative prediction

Given that our method can be applied on single sequence based predictor and can improve its performance. It is interesting to investigate a tricky case, whether our method can be iteratively applied to further improve the prediction performance, as once a sequence based predictor was integrated into our method, the whole predictor can be integrated again as if it is another sequence based predictor.

The iteration process was carried out as follows. In the first round of iteration, the method was identical to all the above, integrating two sequence based predictors. From the second round of iteration, the output from the last round would be used as the only integrated predictor in the method. Table 3 shows the prediction performance of the first four rounds. From the second round of iteration, the prediction performance would not change anymore. The performance in the second round of iteration is slightly better than the first round. This result is expected as the protein-protein interaction network should not be able to improve the prediction performance without a limitation. Because the iterative predictions actually use the information of indirect neighbors in the protein-protein interaction network, this result also implies that it is unnecessary to consider the indirect neighbors in our method.

**Table 3:** Performances of iterative prediction.

Iterations	AIM^a^	CVR^b^	ACC^c^	ATR^d^	AFR^e^
1	79.8%	74.9%	70.0%	56.0%	6.5%
2	80.0%	74.8%	70.0%	56.2%	6.5%
3	80.0%	74.8%	70.0%	56.2%	6.5%
4	80.0%	74.8%	70.0%	56.2%	6.5%

a AIM is Aiming, as defined in eqn (15);

b CVR is Coverage, as defined in eqn (16);

c ACC is Accuracy, as defined in eqn (17);

d ATR is Absolute-True-Rate, as defined in eqn (18);

e AFR is Absolute-False-Rate, as defined in eqn (19).

### 6 Some remarks on the current method

**Figure 3:**
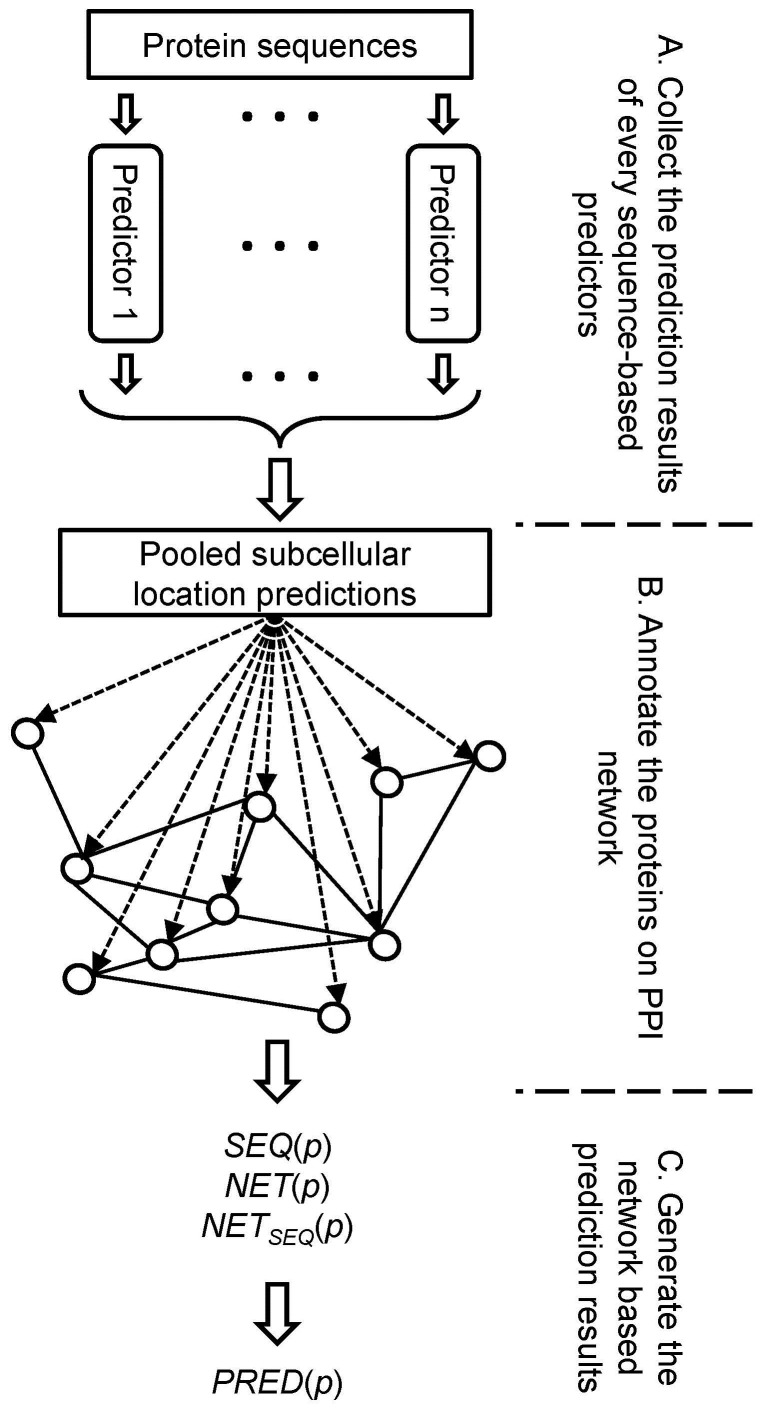
The information flow chart of the whole framework. The input of the framework is only the protein sequences. There are three phases in the whole process. (A) In the first phase, several existing sequence-based predictors give prediction results using only protein sequences. In the current study, these sequence-based predictors include the Y-Loc predictor and the Hum-mPLoc 2.0 predictor. The number *n* is 2. (B) In the second phase, the prediction results of the first phase were collected and then annotated on a protein-protein interaction network. (C) In the third phase, the annotated protein-protein interaction network was analyzed and the network-based prediction results were generated.

Now, let us have a global view of the framework. We have illustrated an information flow chart in Figure 3. The input of the whole framework is only the protein sequences. There are three phases of the whole process. In the first phase, several sequence-based predictors, like Y-Loc and Hum-mPLoc 2.0, make subcellular location predictions. In the second phase, these prediction results are collected and then annotated on a protein-protein interaction network. In the final phase, the annotated protein-protein interaction network is analyzed to produce the final prediction results. Our work focuses only on the second and the third phase, but there is no restriction of what kind of predictors are used in the first phase. Therefore, our method can be used as a wrapper to combine and enhance every existing sequence-based predictor without modifying the predictors themselves. This is the main advantage of current framework.

Although we only treat these sequence-based predictors as black-boxes, which require only protein sequences as input and give subcellular locations as output, we need to remind the readers that some characters of these sequence-based predictors should not be ignored. Some existing sequence-based predictors may use the input protein sequences to generate or to derive other types of features by querying the public databases. For example, the GO annotations, which were generated covertly in some predictors, may cause some potential bias in the results.

In the second phase, there is a problem regarding the protein-protein interaction network. It is well known that the protein subcellular localizations were used as an approach to detect the protein-protein interactions. Therefore, from a view of machine learning, directly using these interactions may cause over-fitting problem. However, this problem does not exist in the current study. Among 96967 protein-protein interactions, there are only 255 interactions that are supported solely by co-localization experiments. These interactions make less than 0.3% of the whole dataset. After manually removing these interactions, there is no observable difference in most of the performance measures. The only observable difference is that the coverage (CVR) increased from 74.9% to 75.0%. Therefore, the risk of over-fitting in the current study can be eliminated.

The final thought regarding this framework is how to characterize the protein-protein interactions. Theoretically, there should be infinite number of measures that could characterize a protein-protein interaction with a number. However, in the current framework, a feasible measure must satisfy the following conditions: (1) it can be calculated solely from the network, as the other types of knowledge may be inconsistent or unavailable to the protein-protein interaction network; (2) it must be highly correlated with the probability that the interacting proteins share subcellular locations; (3) the first two conditions must be satisfied even only incomprehensive and inaccurate protein-protein interaction networks are available, as the knowledge of protein-protein interaction network is still not comprehensive or accurate. As far as we know, the ECC is the only choice we have for this task. Therefore, we believe that, to some extent, ECC is an optimal choice in characterizing the protein-protein interactions in this study.

## Conclusions

In this paper, we proposed a method that can predict protein subcellular locations using protein-protein interaction network as well as the results of existing sequence based predictors. Unlike existing method using a voting scheme to integrate other existing predictors or the other PPI network based methods, the protein-protein interaction network does not provide predictions results all by its own, it works as an infrastructure to coordinate the prediction results of interacting proteins from sequence based predictors.

We applied our method in the human proteome and protein-protein interaction networks. The results shows that our method can improve the sequence based predictions. Since our method can integrate any number of sequence based predictors, this method could serve as a common approach to combine the results of existing methods and improve the prediction performance of almost every existing sequence based methods.
